# Humoral autoimmune response heterogeneity in the spectrum of primary biliary cirrhosis

**DOI:** 10.1007/s12072-012-9413-0

**Published:** 2012-12-05

**Authors:** Alessandra Dellavance, Eduardo Luiz R. Cançado, Clarice Pires Abrantes-Lemos, Michelle Harriz, Valdecir Marvulle, Luis Eduardo C. Andrade

**Affiliations:** 1 Rheumatology Division, Universidade Federal de São Paulo, UNIFESP, Rua Botucatu 740, São Paulo, SP 04023-900 Brazil; 2 Research and Development Division, Fleury Medicine and Health Laboratories, São Paulo, Brazil; 3 Department of Gastroenterology, University of São Paulo School of Medicine, São Paulo, Brazil; 4 Laboratory of Medical Investigation (LIM-06) of Institute of Tropical, Medicine of Sao Paulo University, São Paulo, Brazil; 5 Hospital do Servidor Publico Estadual Francisco Morato de Oliveira, Universidade Federal de Sao Paulo, UNIFESP, São Paulo, Brazil; 6 Statistics Department, Universidade Federal de Sao Paulo, UNIFESP, São Paulo, Brazil

**Keywords:** Autoimmunity, Antibody affinity, Autoimmune liver diseases, Autoantibodies

## Abstract

**Objective:**

To compare autoantibody features in patients with primary biliary cirrhosis (PBC) and individuals presenting antimitochondria antibodies (AMAs) but no clinical or biochemical evidence of disease.

**Methods:**

A total of 212 AMA-positive serum samples were classified into four groups: PBC (definite PBC, *n* = 93); PBC/autoimmune disease (AID; PBC plus other AID, *n* = 37); biochemically normal (BN) individuals (*n* = 61); and BN/AID (BN plus other AID, *n* = 21). Samples were tested by indirect immunofluorescence (IIF) on rat kidney (IIF-AMA) and ELISA [antibodies to pyruvate dehydrogenase E2-complex (PDC-E2), gp-210, Sp-100, and CENP-A/B]. AMA isotype was determined by IIF-AMA. Affinity of anti-PDC-E2 IgG was determined by 8 M urea-modified ELISA.

**Results:**

High-titer IIF-AMA was more frequent in PBC and PBC/AID (57 and 70 %) than in BN and BN/AID samples (23 and 19 %) (*p* < 0.001). Triple isotype IIF-AMA (IgA/IgM/IgG) was more frequent in PBC and PBC/AID samples (35 and 43 %) than in BN sample (18 %; *p* = 0.008; *p* = 0.013, respectively). Anti-PDC-E2 levels were higher in PBC (mean 3.82; 95 % CI 3.36–4.29) and PBC/AID samples (3.89; 3.15–4.63) than in BN (2.43; 1.92–2.94) and BN/AID samples (2.52; 1.54–3.50) (*p* < 0.001). Anti-PDC-E2 avidity was higher in PBC (mean 64.5 %; 95 % CI 57.5–71.5 %) and PBC/AID samples (66.1 %; 54.4–77.8 %) than in BN samples (39.2 %; 30.9–37.5 %) (*p* < 0.001). PBC and PBC/AID recognized more cell domains (mitochondria, nuclear envelope, PML/sp-100 bodies, centromere) than BN (*p* = 0.008) and BN/AID samples (*p* = 0.002). Three variables were independently associated with established PBC: high-avidity anti-PDC-E2 (OR 4.121; 95 % CI 2.118–8.019); high-titer IIF-AMA (OR 4.890; 2.319–10.314); antibodies to three or more antigenic cell domains (OR 9.414; 1.924–46.060).

**Conclusion:**

The autoantibody profile was quantitatively and qualitatively more robust in definite PBC as compared with AMA-positive biochemically normal individuals.

## Introduction

Autoantibodies are hallmarks of autoimmune diseases (AIDs), and disease-specific autoantibodies are valuable diagnostic tools [1]. Primary biliary cirrhosis (PBC) is an autoimmune liver disease involving predominantly intrahepatic biliary duct epithelial cells [2]. Antimitochondria antibodies (AMAs) are detected in roughly 95 % of PBC patients, with a specificity of at least 98 % [3, 4]. AMAs are directed to components of the inner mitochondrial multienzyme 2-oxoacid dehydrogenase complex (2-OADC) implicated in the mitochondrial respiratory chain [5]. The E2 subunit of pyruvate DC (PDC-E2) is the main antigenic moiety in 2-OADC [6]. One-third of PBC patients present a positive result in the antinuclear antibody (ANA) indirect immunofluorescence (IIF) assay on HEp-2 cells (ANA-HEp-2). Two autoantibodies detected in the ANA-HEp-2 assay appear to be specific for PBC and are observed in approximately 25 % of the patients. Anti-gp210 antibodies recognize a nuclear pore glycoprotein [7] and anti-Sp-100 antibodies recognize a 53-kDa protein restricted to the promyelocytic leukemia (PML) nuclear bodies [8]. Anticentromere antibodies have also been observed in PBC, with an apparent association with portal hypertension [9, 10].

**Fig. 1:**
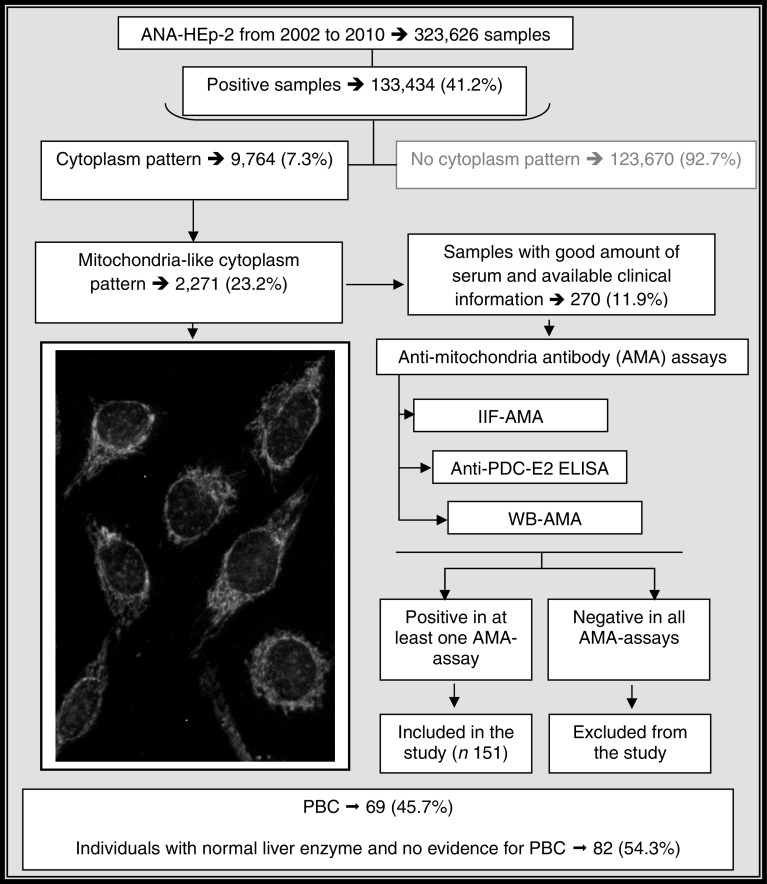
Algorithm for selection of samples according to the identification of the peculiar cytoplasm mitochondria-like pattern in the antinuclear antibody (ANA) indirect immunofluorescence (IIF) assay on HEp-2 cells assay

The ANA-HEp-2 test is the standard screening assay for autoantibodies [11], and the immunofluorescence pattern in a positive ANA-HEp-2 test may indicate the possible autoantibody specificities present in a given sample [11–13]. AMA yields a peculiar cytoplasm pattern in the ANA-HEp-2 assay (Fig. 1). By processing roughly 323,000 samples in the ANA-HEp-2 assay in a time frame of 8 years, we identified a considerable number of samples with the characteristic AMA-like cytoplasm pattern (Fig. 1). Processing such samples in AMA-specific assays eventually resulted in a sizable number of AMA-positive asymptomatic individuals with normal liver enzyme serum levels (Fig. 1). The significance of such AMA response in apparently normal individuals is unknown. Neither is it known how this immune response compares with the autoimmune response observed in clinically established PBC?

The intrinsic features of any given humoral response are quite heterogeneous with respect to titer, avidity, and immunoglobulin isotype of the antibodies, as well as the spectrum of targeted antigens and epitopes. In the present study we investigated AMA serum levels, AMA isotypes, and the avidity and serum levels of anti-PDC-E2 IgG antibodies in PBC patients and in AMA-positive asymptomatic and biochemically normal individuals. Reactivity against other mitochondrial antigens and the serum levels of antibodies to gp210, Sp-100, and the centromere proteins CENP-A and CENP-B were also investigated.

## Materials and methods

The 212 AMA-positive samples used in the present study were derived from two sources: 61 (28.7 %) samples from patients with definite PBC attending at the Hepatology Division of Sao Paulo University School of Medicine and 151 (71.2 %) samples selected from the ANA-HEp-2 daily operation from January 2002 to January 2010 at Fleury Medicine and Health Laboratories (certified on-site by the College of American Pathologists/CAP). Throughout these 8 years, 323,626 samples were processed for ANA-HEp-2. Samples were suspected to be AMA-positive because of the AMA-like pattern in the ANA-HEp-2 assay (Fig. 1). Those confirmed to be AMA-positive on specific assays (IIF on rodent tissue, ELISA, or Western blot) were selected for the present study. Clinical data were obtained by chart review and interview with the physicians who ordered the tests. PBC diagnosis was established according to the American Association for Study of Liver Diseases criteria [14]. For most patients consistently increased alkaline phosphatase serum levels and AMA were enough to establish PBC diagnosis. Some patients required liver biopsy for confirmation. Whenever appropriate, image studies were undertaken to rule out the possibility of biliary tract obstruction. Serum liver enzymes were determined in all samples of patients without definite PBC diagnosis. Samples were classified into four groups: PBC group (*n* = 93)—definite PBC according to established diagnostic criteria [14]; PBC/AID group (*n* = 37)—definite PBC plus any nonhepatic AID; biochemically normal (BN) group (*n* = 61)—individuals with no apparent disease and normal alkaline phosphatase serum levels; and BN/AID group (*n* = 21)—BN plus any nonhepatic AID. BN and BN/AID individuals had at least two samples separated by at least 6 months with normal alkaline phosphatase levels. Samples with slightly elevated alkaline phosphatase serum levels (less than twofold the upper normal limit) and no further evidence of PBC were excluded from the present study. Samples were processed without the knowledge of their identity and the group to which they belonged. The study was approved by the National Committee for Ethics in Research (CONEP).

AMA was determined by IIF (IIF-AMA) and ELISA. IIF-AMA on in-house rodent tissue preparations was performed as described elsewhere [15]. Samples were screened at 1:40 and serially diluted up to end-point fluorescence or to 1:2,560. Positive samples were tested for AMA isotype with fluorescein isothiocyanate (FITC)-conjugated goat antibodies against human IgG (BioMérieux, Marcy l’Etoile, France), IgM and IgA (Dako, Bucks, UK) at 1:200 (anti-IgG and anti-IgM) and 1:20 (anti-IgA). ANA-HEp-2 was performed at 1:160 in HEp-2 cell slides (Bion, Des Plaines, IL, USA) following the manufacturer’s instructions. In addition to the characteristic cytoplasm mitochondria-like pattern, we registered the occurrence of reactivity to the nuclear envelope, multiple nuclear dots, and centromere. Slides were independently analyzed by two blinded readers (AD and LECA) using an Olympus B50 fluorescence microscope (Center Valley, PA USA) at ×400 magnification.

Reactivity against purified gp210 and Sp-100, and recombinant CENP-A/B was determined by ELISA (INOVA Diagnostics), according to the manufacturer’s instructions. Antibodies against PDC-E2 (M2 fraction) were determined by ELISA (Orgentec, Mainz, Germany) according to the manufacturer’s instructions. The affinity of anti-PDC-E2 IgG was determined under chaotropic conditions [16]. Samples were incubated in quadruplicate in the standard anti-PDC-E2 ELISA for 1 h. Next, for each quadruplicate set, two wells were incubated with regular washing solution and two wells were incubated with urea 8 M in PBS-T for 15 min at room temperature. Plates were then washed and further processed as per the regular ELISA. Affinity was estimated by dividing the optical density observed in the wells submitted to urea treatment by the optical density of wells without urea treatment.

IgG reactivity against individual 2-OADC antigens [E2 subunits of PDC-E2/74 kDa, BCOADC (56 kDa), OGDC (52 kDa), and the E3BP (36 kDa), E1alpha (48 kDa), E1-beta subunits (41 kDa)] was determined by Western blot (WB) [17–19]. Briefly, mitochondria-rich liver extract (10 mg/mL) was separated by 10 % sodium dodecyl sulfate–polyacrylamide gel electrophoresis and transferred onto nitrocellulose filters. After blocking in PBS containing 5 % skim milk for 2 h, longitudinal strips were incubated with individual serum samples diluted 1:50 in blocking solution for 1 h. Strips were then washed three times in PBS-T and incubated for 1 h at room temperature in horseradish peroxidase-conjugated rabbit anti-human IgG 1:1,500 (Bio-Rad, Hercules, CA, USA) in blocking solution. After washing in PBS-T, the colorimetric reaction was developed for 10 min in 6 mg 4-chloro-1-naphthol diluted in 2 mL methanol added to 10 mL PBS containing 20 μL 30 % H_2_O_2_. The reaction was stopped with distilled water after the development of bands.

Categorical variables were analyzed by the Chi-square test. Kruskal–Wallis and Mann–Whitney tests were used to compare quantitative and semiquantitative nonparametric variables. Multiple regression analysis was used to identify variables independently associated with the classification as definite PBC. The nomogram model was used to calculate the interaction of the independent variables. A *p* value of less than 0.05 was considered significant.

## Results

The majority of individuals were females (90.6 %), and there was no significant difference in gender and age distribution among the four groups (Table 1). Information on current liver enzyme serum levels was available for all BN and BN/AID subjects, but not for all PBC and PBC/AID patients. Increased alkaline phosphatase serum levels were observed at the time of the study in 37 % of PBC patients and in 25 % of PBC/AID patients. Normal alkaline phosphatase levels in PBC and PBC/AID groups were because of ursodeoxycholic acid therapy, which was used in the majority of these patients (Table 1). Liver biopsy information was available for one-quarter of PBC patients and half of PBC/AID patients, most of whom exhibited lesions compatible with stages II and III (Table 1).

**Table 1:** Demographic and clinical characteristics of the 212 studied individuals

Parameters	Groups			
	PBC (*n* = 93)	PBC/AID (*n* = 37)	BN (*n* = 61)	BN/AID (*n* = 21)
No. of females* (%)	84 (90.3 %)	32 (86.5 %)	57 (93.4 %)	19 (90.5 %)
Age (years)^**,a^	54.2 (29–85)	51.6 (31–73)	51.5 (26–84)	49.5 (18–68)
Biochemistry and therapeutics				
Alkaline phosphatase^a^	229 (52–776)	231 (117–385)	67 (32–115)	59 (16–99)
Increased alkaline phosphatase	23/61 (37.7 %)	2/8 (25 %)	0/61	0/21
Ursodeoxycholic acid^b^	65/85 (76.4 %)	30/36 (83.3 %)	0/61	0/21
Liver biopsy				
Biopsy available	25/93 (26.8 %)	21/37 (56.7 %)	Not applicable	
Stage I	2/25 (8 %)	2/21 (9.5 %)		
Stage II	5/25 (20 %)	7/21 (33.3 %)		
Stage III	11/25 (44 %)	8/21 (38 %)		
Stage IV	4/25 (16 %)	3/21 (14.2 %)		
Nonconclusive	3/25 (12 %)	1/21 (4.7 %)		
Associated diseases				
CREST	0	7 (19 %)	0	0
Systemic sclerosis	0	1 (2.7 %)	0	1 (4.7 %)
Sjögren’s syndrome	0	12 (32.4 %)	0	2 (9.5 %)
Rheumatoid arthritis	0	4 (10.8 %)	0	2 (9.5 %)
Celiac disease	0	4 (10.8 %)	0	0
SLE	0	1 (2.7 %)	0	0
Thyroid disease	0	2 (5.4 %)	0	2 (9.5 %)
Miscellaneous^c^	0	12 (32.4 %)	0	16 (76.1 %)

Reference values for alkaline phosphatase (female 35–104 U/mL, male 40–129 U/mL)

*BN* biochemically normal individuals, *BN/AID* biochemically normal individuals with some other autoimmune disease, *PBC* primary biliary cirrhosis, *PBC/AID* primary biliary cirrhosis plus other autoimmune disease, *SLE* systemic lupus erythematosus

* *p* = 0.725; ** *p* = 0.383

^a^Mean and range

^b^Number of patients receiving ursodeoxycholic acid over the number of patients with information about therapeutic regimen

^c^Miscellaneous: psoriasis, pulmonary vasculitis, polymyositis, pemphigus, autoimmune retinitis, renal tubular acidosis, Raynaud phenomenon, temporal arteritis, polymyalgia rheumatica, psoriatic arthritis, seronegative polyarthritis, colon diverticulitis, isolated sacroiliitis, non-specific muscle-skeletal pain, pernicious anemia, idiopathic trombocytopenic purpura

**Fig. 2:**
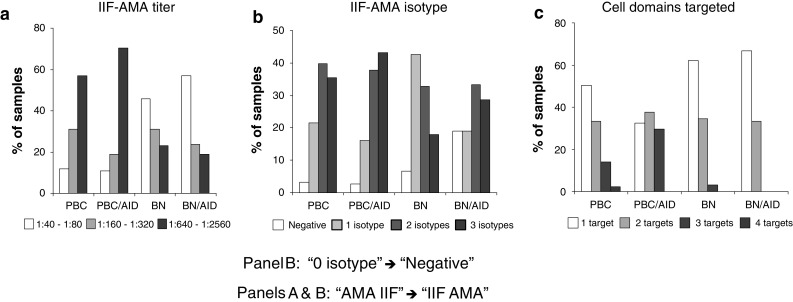
Distribution of samples according to **a** indirect immunofluorescence (IIF) on rat kidney (IIF-AMA) titer (*p* < 0.001), **b** number of IIF-AMA isotypes (*p* = 0.005), **c** number of antigenic cellular domains targeted (*p* = 0.005). BN, AMA-reactive individuals with normal levels of alkaline phosphatase; BN/AID, same as BN but with any associated extrahepatic autoimmune disease; PBC, definite primary biliary cirrhosis; PBC/AID, definite primary biliary cirrhosis and any associated extrahepatic autoimmune disease

IIF-AMA was positive in 94.3 % (200/212) of the samples (Table 2) and the remaining 12 samples were reactive in PDC-E2 ELISA or WB-AMA. IIF-AMA frequency was slightly lower in the BN/AID group (*p* = 0.021). IIF-AMA titers were arbitrarily divided into low (1:40–1:80), medium (1:160–1:320), and high strata (1:640–1:2,560). High titer IIF-AMA was associated with PBC and PBC/AID groups, whereas low titer IIF-AMA was associated with BN and BN/AID groups (Fig. 2a). In addition, there were some differences in IIF-AMA isotype among the groups (Table 2). All IIF-AMA-positive samples had the IgG isotype. IgM IIF-AMA was more frequent in PBC and PBC/AID than in the BN group (*p* = 0.003 and *p* = 0.004, respectively). IgA IIF-AMA was also more frequent in PBC and PBC/AID than in the BN group (*p* = 0.023 and *p* = 0.007, respectively). Finally, PBC and PBC/AID had a higher frequency of samples with triple isotype IIF-AMA than the BN group (*p* = 0.013 and *p* = 0.008, respectively) (Table 2; Fig. 2b).

**Table 2:**
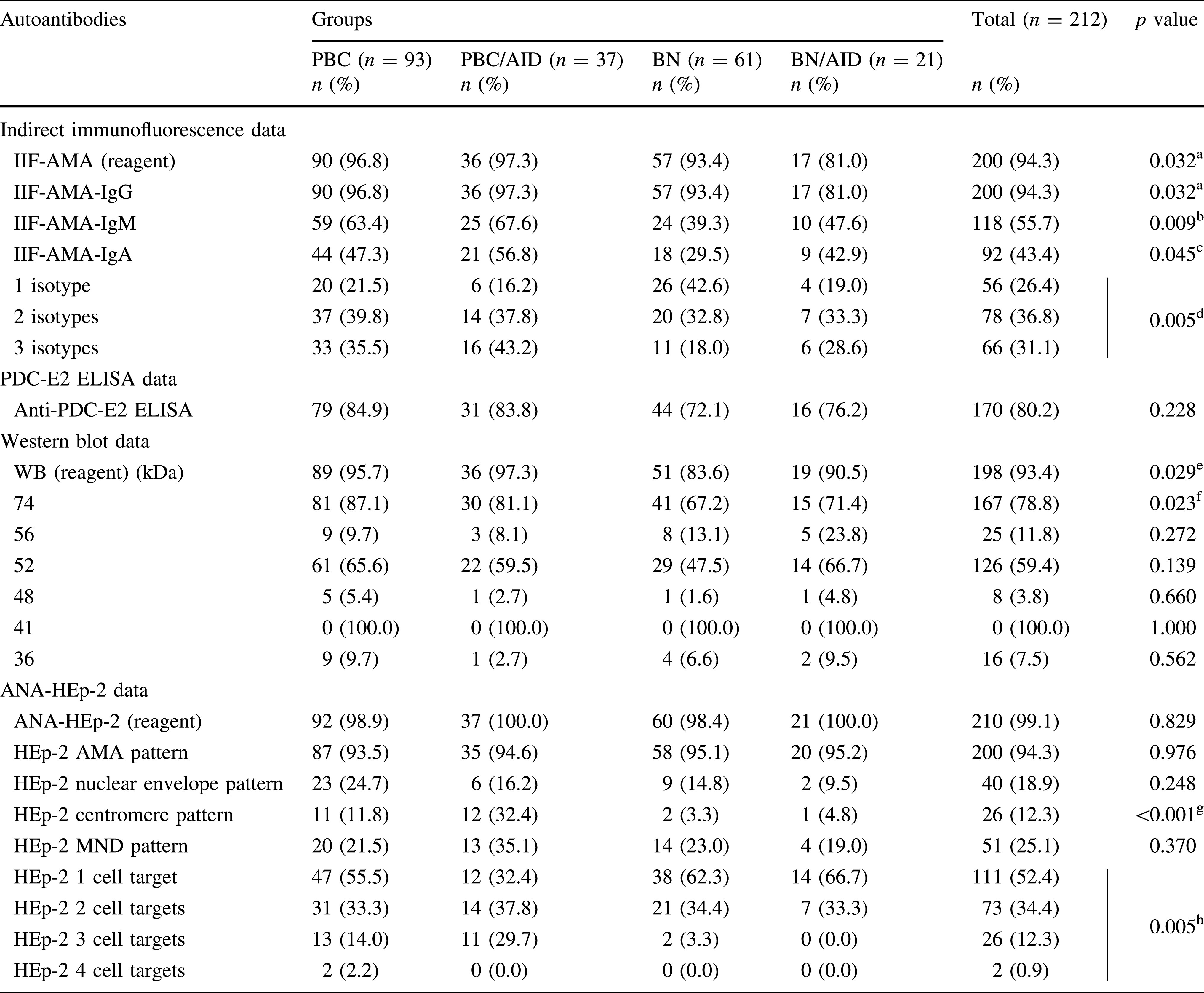
Distribution of AMA-positive samples of primary biliary cirrhosis patients and biochemically normal individuals according to the reactivity in multiple assays for autoantibodies

*BN* biochemically normal individuals, *BN/AID* biochemically normal individuals with some other autoimmune disease, *PBC* primary biliary cirrhosis, *PBC/AID* primary biliary cirrhosis plus other autoimmune disease

^a^BN/AID × PBC (*p* = 0.021)

^b^PBC × BN (*p* = 0.003); PBC/AID × BN (*p* = 0.004)

^c^PBC × BN (*p* = 0.023); PBC/AID × BN (*p* = 0.007)

^d^1 isotype: BN × PBC (*p* = 0.006) and BN × PBC/AID (*p* = 0.003); 3 isotypes: PBC × BN (*p* = 0.013) and PBC/AID × BN (*p* = 0.008)

^e^BN × PBC (*p* = 0.019) and BN × PBC/AID (*p* = 0.048)

^f^PBC × BN (*p* = 0.004)

^g^PBC/AID × PBC (*p* = 0.014); PBC/AID × BN (*p* < 0.001); PBC/AID × BN/AID (*p* = 0.021)

^h^1 cellular target: BN/AID × PBC/AID (*p* = 0.008); BN × PBC/AID (*p* = 0.003); ≥3 cellular targets PBC/AID × BN (*p* < 0.001); PBC/AID × BN/AID (*p* = 0.005); PBC × BN (*p* = 0.048)

**Fig. 3:**
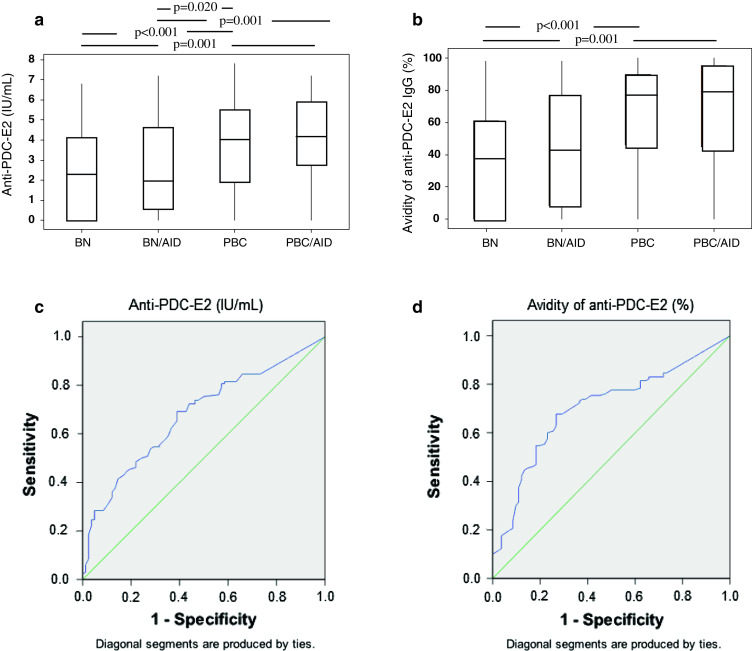
Distribution of samples according to **a** titer concentration (IU/mL) of anti-PDC-E2 IgG (*p* < 0.001) and **b** avidity of anti-PDC-E2 IgG (*p* < 0.001). Receiver operating curve analysis comparing PBC and PBC/AID samples versus BN and BN/AID samples according to **c** titer concentration (IU/mL) of anti-E2-PDC IgG [AUC = 0.679 (95 % CI 0.606–0.751)] and **d** avidity of anti-E2-PDC IgG [AUC = 0.704 (95 % CI 0.633–0.755)]. BN, AMA-reactive asymptomatic individuals with normal levels of alkaline phosphatase; BN/AID, same as BN but with any associated extrahepatic autoimmune disease; PBC, definite primary biliary cirrhosis; PBC/AID, definite primary biliary cirrhosis and any associated extrahepatic autoimmune disease

The four groups were equivalent in the frequency of positive samples for anti-PDC-E2 (Table 2). However, PBC and PBC/AID groups presented higher serum levels of anti-PDC-E2 antibodies than BN and BN/AID groups (Fig. 3a). There was no difference in serum levels of anti-PDC-E2 antibodies between PBC and PBC/AID, and between BN and BN/AID groups. Receiver operating curve (ROC) analysis indicated that the best anti-PDC-E2 serum level to discriminate PBC and PBC/AID from BN and BN/IAD groups was 3.0 IU/mL (Fig. 3c). The avidity of anti-PDC-E2 IgG was determined in all anti-PDC-E2-reactive samples (170/212) and was found to be significantly higher in PBC and PBC/AID than in the BN group (Fig. 3b). The best anti-PDC-E2 IgG avidity level to discriminate PBC and PBC/AID from BN and BN/IAD groups was 64 % (Fig. 3d). WB also showed some differences among the four groups (Table 2). The BN group had a lower frequency of reactive samples than PBC (*p* = 0.019) and PBC/AID (*p* = 0.048) groups. In addition, the PBC group presented a higher frequency of 74-kDa reactive samples than the BN group (*p* = 0.004) (Table 2).

**Fig. 4:**
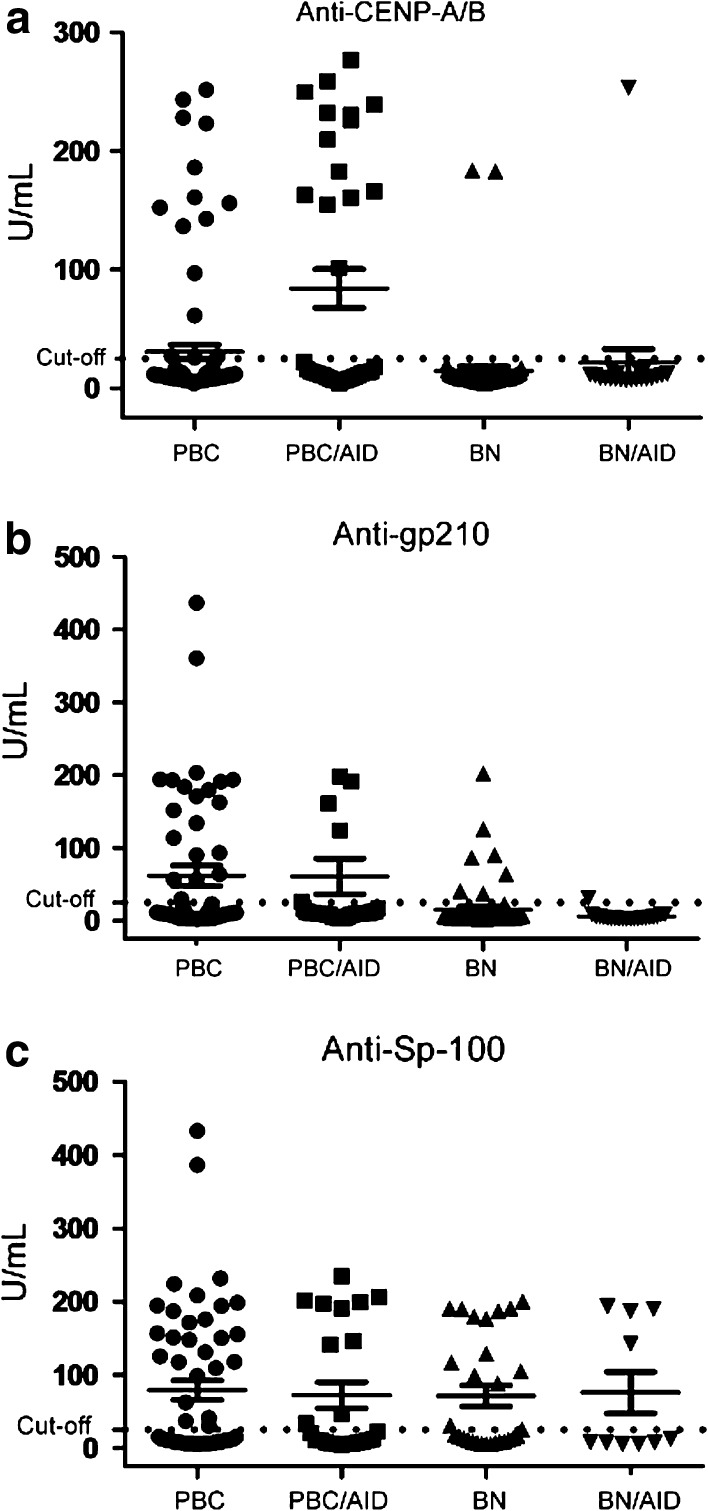
Distribution of samples according to results of ELISA tests. **a** Anti-CENP-A/B antibody serum levels presented higher values in PBC/AID samples as compared with PBC (*p* = 0.006), BN (*p* < 0.001), and BN/AID samples *(p* = 0.030). **b** Anti-gp210 antibody serum levels were higher in PBC and PBC/AID groups as compared with that in BN and BN/AID groups (*p* = 0.0032). **c** Anti-Sp-100 antibody serum levels showed no difference among the groups of samples (*p* = 0.808). BN, AMA-reactive asymptomatic individuals with normal levels of alkaline phosphatase; BN/AID, same as BN but with any associated extrahepatic autoimmune disease; PBC, definite primary biliary cirrhosis; PBC/AID, definite primary biliary cirrhosis and any associated extrahepatic autoimmune disease

Concerning the ANA-HEp-2 patterns indicative of specific cell domains (centromeres, nuclear envelope, and PML bodies), the centromere pattern was significantly more frequent in PBC/AID samples than in PBC (*p* = 0.014), BN (*p* < 0.001), and BN/AID samples (*p* = 0.021) (Table 2). Anti-CENP-A/B antibodies were detected in 31 of the 212 samples with a higher frequency in PBC/AID (14/37; 37.9 %) than in PBC (14/93; 15.1 %; *p* = 0.009), BN (2/61; 3.3 %; *p* < 0.001), and BN/AID groups (1/21; 4.8 %; *p* = 0.014). Anti-CENP-A/B antibodies presented higher serum levels in PBC/AID than in PBC (*p* = 0.006), BN (*p* < 0.001), and BN/AID samples (*p* = 0.030) (Fig. 4a). The 14 PBC/AID patients with anticentromere antibodies had varied diagnoses (41.6 % CREST syndrome, 33.3 % Sjögren syndrome, 16.6 % Raynaud syndrome, and 8.3 % celiac disease), for which the very presence of anticentromere antibodies was not a driving diagnostic criterion. There was no significant difference regarding other ANA-HEp-2 patterns among the four groups (*p* = 0.248) (Table 2). However, the number of cell antigenic domains (mitochondria, nuclear envelope, PML/Sp-100 domain, and centromere) recognized per individual sample differed among the four groups (*p* = 0.005). PBC and PBC/AID groups presented a higher frequency of samples recognizing three or more targets as compared with BN (*p* = 0.048 and <0.001, respectively) (Table 2; Fig. 2c).

The frequency of anti-gp210 antibodies was equivalent among the four groups but anti-gp210 serum levels were higher in PBC and PBC/AID groups (Fig. 4b). In fact, anti-gp210 levels above 100 AU/mL were observed in 16 (12.9 %) of the PBC and PBC/AID samples but only 1 (1.2 %) of the BN and BN/AID samples (*p* = 0.0032). Anti-Sp-100 antibodies showed a similar frequency in all samples presenting any definite nuclear fluorescence in the ANA-HEp-2 test [PBC: 23 of 53 (43.4 %); PBC/AID: 12 of 24 (50 %); BN: 13 of 30 (43.3 %); and BN/AID: 4 of 10 (40 %)] (*p* = 0.808). There was no difference in serum levels of anti-Sp-100 antibodies among the four groups (Fig. 4c).

Several serological parameters presented a significant association with the current classification of samples as belonging to patients with definite PBC (Table 3), including high titer IIF-AMA, triple isotype IIF-AMA, high titer and high avidity anti-PDC-E2 antibodies, and three or more cellular domains recognized by autoantibodies. Multiple regression analysis identified three independent variables presenting a significant association with definite PBC: high avidity anti-PDC-E2 antibodies (OR 4.12; 95 % CI 2.12–8.02), high titer IIF-AMA antibodies (OR 4.89; 95 % CI 2.32–10.31), and three or more cell domains recognized by autoantibodies (OR 9.41; 95 % CI 1.92–46.06). These three independent variables interact with each other to further increase the probability of a current diagnosis of PBC (Table 4).

**Table 3:** Performance of parameters of the humoral autoimmune response according to their ability to classify the 212 samples as associated with definite primary biliary cirrhosis (PBC or PBC/AID)

Serologic parameter	Ranges	Classification as PBC or PBC/AID					
		Se	Sp	PPV	NPV	Accuracy	OR (95 % CI)
		(Values expressed in percentage)					
IIF-AMA titer	Low (0–1/80)	13.1	52.4	30.4	27.6	28.3	0.72 (0.174–4.12)
	(1/160–1/320)	26.9	69.5	58.3	37.5	43.4	4.00 (1.82–8.79)
	High (≥1/640)	60.0	79.3	82.1	55.6	67.5	11.70 (5.35–25.63)
IIF-AMA isotypes	Negative or 1	23.1	53.7	44.1	30.6	34.9	1.73 (0.47-6.43)
	2	39.2	67.1	65.4	41.0	50.0	3.77 (1.04–13.69)
	3	37.7	79.3	74.2	44.5	54.0	5.76 (1.54–21.60)
Anti-PDC-E2 levels^a^	≥3.0 IU/mL	68.5	61.0	73.6	55.0	65.6	3.51 (1.97–6.27)
Anti-PDC-E2 avidity^b^	≥64 %	67.7	73.2	80.0	58.8	69.8	5.71 (3.10–10.53)
WB-AMA	≥2 proteins	61.5	57.3	69.6	48.5	59.9	2.14 (1.22–3.77)
Antigenic cell domains	≥3 targets	20.0	97.6	92.9	43.5	50.0	10.00 (2.31–43.38)

*Se* sensitivity, *Sp* specificity, *PPV and NPV* positive and negative predictive value, respectively, *OR* odds ratio, *95* *% CI* 95 % confidence interval, *WB-AMA* Western blot for antimitochondria antibodies

^a^ROC curve for anti-PDC-E2 serum levels, AUC = 0.679 (0.606–0.751)

^b^ROC curve for anti-PDC-E2 avidity, AUC = 0.704 (0.633–0.755)

**Table 4:** Nomogram analysis of the interaction of the three independent variables regarding the probability of classification of samples as definite primary biliary cirrhosis (PBC or PBC/AID)

	Anti-PDC-E2 IgG avidity <64 %		Anti-PDC-E2 IgG avidity ≥64 %	
	Antigenic cellular targets		Antigenic cellular targets	
IIF-AMA titer	≤2	≥3	≤2	≥3
Low	20.5 % (9.3–36.5 %)^a^	66.7 % (9.4–99.1 %)	38.5 % (13.9–68.4 %)	–^b^
Medium or high	50.0 % (36.1–63.9 %)	83.3 % (35.9–99.6 %)	82.1 % (71.7–89.8 %)	100 % (85.4–100.0 %)

*IIF-AMA* indirect immunofluorescence for antimitochondria antibodies

^a^95 % confidence interval in parentheses

^b^Not enough information to perform prevision

## Discussion

The present study disclosed several differences in the intrinsic features of the autoantibody profile in individuals with AMA reactivity and normal levels of alkaline phosphatase as opposed to patients with definite PBC. Patients with definite PBC displayed a more vigorous autoantibody profile, represented by higher serum levels of IIF-AMA, a higher frequency of triple isotype IIF-AMA, higher serum levels and higher avidity anti-PDC-E2 IgG, and higher titer anti-gp210 antibodies. In addition, the autoantibody profile in patients with definite PBC addressed a broader set of antigenic targets, recognizing a higher number of cell domains than individuals with no biochemical or clinical evidence of PBC. These differences were true regardless of the presence of an associated extrahepatic AID. Multiple regression analysis identified three independent risk factors for the classification of a sample as belonging to biochemically normal individuals or to patients with definite PBC, namely high titer IIF-AMA, high avidity anti-PDC-E2 antibodies, and widespread reactivity against multiple cell domains. This observation might be clinically useful in the instance of an unexpected positive AMA result in an individual with no clinical and biochemical evidence for PBC. Obviously, these findings must be confirmed by similar studies in independent series of AMA-reactive biochemically normal samples and by longitudinal studies comparing AMA-positive samples before and after the development of liver involvement.

What is the exact clinical situation of AMA-positive asymptomatic individuals with normal alkaline phosphatase levels? Could they represent preclinical stages of PBC? Could they represent normal individuals with no relationship with the PBC disease spectrum? Because of the design of the present study and the setting in which samples were obtained, one can determine that they had no clinical or biochemical evidence of PBC at the moment of the study. However, we cannot rule out the possibility that some of them had varied degrees of histological biliary tract involvement typical of PBC and therefore represented preclinical stages of histopathologically established disease. Regardless of the histological status, it is reasonable to admit that some of these individuals will eventually develop definite PBC. In fact, previous follow-up studies of AMA-positive asymptomatic cohorts have shown that a significant proportion of individuals will develop overt disease within a variable time interval [14, 19]. In this context, it is relevant to consider that the 82 AMA-positive and biochemically normal individuals represent 0.02 % of 323,000 individuals screened. This frequency is not far from the estimated prevalence of PBC in the general population [14]. However, because of the cross-sectional design of the present study we cannot determine the fraction of these individuals who will eventually develop overt PBC. With these restrictions in mind we may consider that these individuals might represent a heterogeneous group, comprising potential patients at preclinical stages of PBC and normal subjects with no relationship to PBC. Overall, the obtained data are quite provocative because they shed some light onto the nature of the autoimmune response at very early stages of PBC.

It is well established that disease-specific autoantibodies frequently precede the onset of symptoms and the diagnosis of the cognate diseases for months or years. Examples of such include antinative DNA antibodies and systemic lupus erythematosus [20], anticitrullinated peptide antibodies or rheumatoid factor and rheumatoid arthritis [21], antithyroid peroxidase antibodies and Hashimoto thyroiditis [22], anti-insulin antibodies and type I diabetes mellitus [23], and AMAs and PBC [24]. The consistent demonstration of disease-specific autoantibodies preceding the clinical onset of diverse AIDs seems to indicate that immunological disturbances regularly precede the establishment of overt disease for a variably long preclinical period in which no or very low inflammatory activity is present at the target tissues. Understanding the immunology of such “pre/low-inflammatory” stages of AIDs may allow the development of effective immunomodulatory therapy to successfully prevent the full development of such illnesses. An initial approach to study the immunology of the “pre/low-inflammatory” stages of AIDs is to analyze the intrinsic features of autoantibodies before and after their clinical onset. PBC appears to be an appropriate model for this objective because its natural history comprises a long period in which circulating autoantibodies are detected in the absence of clinical and biochemical events [25]. The relationship between this particular autoimmune response and the etiology of PBC as well as the precise mechanisms of bile duct destruction remains unclear, and some authors believe that autoantibodies represent an epiphenomenon not directly related to the disease pathophysiology. The findings of the present study suggest that along the transition from early to later stages of PBC there are marked qualitative and quantitative changes in the autoantibody profile represented by a higher rate of production and higher avidity of AMA, as well as the spreading of antigenic targets recognizing several cell domains. Although the methodological restrictions of the present study preclude definite conclusions, these preliminary findings encourage follow-up studies to determine the longitudinal behavior of the humoral autoimmune response along the transition from early to later stages of PBC.

In summary, the present study has provided evidence for marked qualitative and quantitative differences in the autoantibody profile of AMA-positive individuals with normal alkaline phosphatase levels and patients with established PBC. The present data indicate that high titer AMA, high avidity anti-PDC-E2 antibodies, and widespread response to multiple cell domains represent risk factors for a given AMA-positive sample to be associated with definite PBC. This is an original finding that may shed some light onto the understanding of the obscure immunological abnormalities preceding the instatement of the full-blown inflammatory stage of PBC and other AIDs. Studies on patients with different PBC histological stages and AMA-positive individuals with no biochemical or histological evidence of liver disease are required to validate these preliminary results.
